# Enhanced Self-Checkout System for Retail Based on Improved YOLOv10

**DOI:** 10.3390/jimaging10100248

**Published:** 2024-10-10

**Authors:** Lianghao Tan, Shubing Liu, Jing Gao, Xiaoyi Liu, Linyue Chu, Huangqi Jiang

**Affiliations:** 1 Department of Information System, Arizona State University, Tempe, AZ 85281, USA; 2 Department of Computer Science, University of North Carolina, Chapel Hill, NC 27599, USA; 3 Department of Industrial and Systems Engineering, University of Minnesota, Minneapolis, MN 55455, USA; 4 Ira A. Fulton Schools of Engineering, Arizona State University, Tempe, AZ 85281, USA; 5 Henry Samueli School of Engineering, University of California, Irvine, CA 92617, USA; 6 Department of Psychological Sciences, Case Western Reserve University, Cleveland, OH 44106, USA

**Keywords:** YOLO, self-checkout system, computer vision, retail

## Abstract

With the rapid advancement of deep learning technologies, computer vision has shown immense potential in retail automation. This paper presents a novel self-checkout system for retail based on an improved YOLOv10 network, aimed at enhancing checkout efficiency and reducing labor costs. We propose targeted optimizations for the YOLOv10 model, incorporating the detection head structure from YOLOv8, which significantly improves product recognition accuracy. Additionally, we develop a post-processing algorithm tailored for self-checkout scenarios, to further enhance the application of the system. Experimental results demonstrate that our system outperforms existing methods in both product recognition accuracy and checkout speed. This research not only provides a new technical solution for retail automation but offers valuable insights into optimizing deep learning models for real-world applications.

## 1. Introduction

### 1.1. Research Background

Efficient retail operations are critical to ensuring customer satisfaction and maintaining competitive advantage in the fast-paced market today. Self-checkout systems, in particular, have become an essential component of modern retail environments, offering customers a quicker, more convenient way to complete their purchases [1]. These systems help reduce the burden on staff, minimize wait times, and enhance the overall shopping experience. As consumer expectations for speed and convenience rise, the demand for reliable and accurate self-checkout solutions continues to grow. However, the effectiveness of these systems depends on their ability to accurately identify and process a diverse range of products in real time, a challenge that has driven significant research and development in the field of computer vision and deep learning.

### 1.2. Challenges in Current Self-Checkout Systems

Despite the growing adoption of self-checkout systems in modern retail environments, several challenges persist that can hinder their effectiveness. One of the primary issues is the accurate recognition and differentiation of a wide variety of products, especially in environments with complex backgrounds, varying lighting conditions, or occluded items [2]. Traditional systems often struggle with identifying items that are similar in appearance or those that are irregularly shaped, leading to increased error rates and customer frustration. Additionally, maintaining real-time processing capabilities without compromising accuracy remains a significant hurdle, particularly as the volume of transactions and the diversity of products continue to expand [3]. Handling overlapping objects presents a significant challenge, as current self-checkout systems often struggle when products are partially occluded or stacked, which is a common occurrence when customers place multiple items on the checkout surface. Additionally, these systems face difficulties with sub-optimal projections, particularly when products are positioned irregularly or exposed to varying lighting conditions. Such scenarios can severely compromise recognition accuracy and hinder the overall efficiency of the checkout process [4]. These challenges underscore the need for ongoing research and development in computer vision and deep learning to create more robust and reliable self-checkout systems that can meet the demands of today’s retail industry.

### 1.3. Overview of Advancements of Deep Learning and Computer Vision in Retail

With the rapid advancement of technology, the wave of artificial intelligence (AI) is profoundly transforming a wide range of industries, including retail [5,6]. Both 2D and 3D vision models are widely used in different scenarios [7,8], and real-time tracking detection can be used on both manned and unmanned objects [9,10]. In the realm of AI, machine learning (ML) has become a powerful tool for tackling complex tasks and enhancing efficiency. Deep learning (DL) and computer vision (CV), two significant subsets of ML, have gained attention in recent years for their precise handling and analysis of visual data even in difficult and remote scenarios [11,12].

The development of powerful convolutional neural networks (CNNs) and real-time object detection algorithms, such as the You Only Look Once (YOLO) series, has enabled machines to process visual data with unprecedented speed and accuracy [13,14]. These technologies have greatly improved the ability to recognize and classify retail products in various environments, even under challenging conditions such as occlusions, varying lighting, and complex backgrounds.

### 1.4. Purpose of the New System and Advantages

The purpose of the current study is to develop an enhanced self-checkout system that effectively addresses the limitations of existing technologies by leveraging the latest advancements in deep learning and computer vision. Our proposed system, MidState-YOLO-ED, integrates key innovations from YOLOv10 with enhancements inspired by YOLOv8, aiming to significantly improve product recognition accuracy and processing efficiency in real-time retail environments [15,16]. The advantages of this system include its ability to accurately identify a diverse range of retail products, maintain robust performance under challenging conditions, and operate efficiently on resource-constrained devices. These improvements are designed to make the self-checkout process faster, more reliable, and better suited to the demands of modern retail settings.

## 2. Related Work

### 2.1. Traditional Object Detection Algorithms

Object detection has long been a central challenge in computer vision, with traditional approaches primarily relying on CNNs to identify and locate objects within images. Among the most notable early models are Faster R-CNN, Single Shot MultiBox Detector (SSD), and the original YOLO series. Before the advent of DL, the field of object detection primarily relied on traditional manual feature engineering techniques, while DL advanced the field of object detection and brought its benefit to retail operators and consumers [17,18]. Solutions from this era typically followed a two-stage process: feature extraction and classification. Besides the advancement of algorithms for exploring data samples [19,20], techniques from image processing and computer vision were used to manually construct and extract prominent features from images, such as Haar feature sets and scale-invariant feature transform (SIFT) [21]. These designed features were then fed into classifiers to identify objects within images. However, the effectiveness of this approach heavily depended on the quality of the feature engineering and often fell short in complex and variable image backgrounds and scenes.

With the widespread adoption of CNNs, a significant leap occurred in object detection. CNNs, with their robust automatic feature-learning capabilities, eliminated the need for cumbersome manual feature design [22,23]. They could directly learn hierarchical and rich feature representations from raw image data, greatly enhancing the accuracy and efficiency of object detection. Faster R-CNN marked a significant milestone by introducing the region proposal network (RPN), which efficiently generates region proposals that are likely to contain objects, reducing the need for exhaustive search methods and improving detection accuracy [24]. However, its two-stage process, which separates region proposal and classification, makes it computationally intensive, limiting its real-time application.

SSD aimed to balance accuracy and speed by eliminating the region proposal stage entirely. Instead, it predicts object classes and bounding boxes directly from feature maps at different scales, allowing for the detection of objects of various sizes in a single pass [25]. While SSD offers improved speed over Faster R-CNN, it still struggles with detecting smaller objects and achieving the highest levels of accuracy [26].

RT-DETR (real-time detection transformer) aims to optimize key components of DETR, achieving real-time and high-precision object detection [27]. It represents an innovative real-time end-to-end object detection model. The RT-DETR model consists of three components: the backbone network, a hybrid encoder, and a transformer decoder equipped with an auxiliary prediction head. The backbone network uses convolutional networks to extract salient features at three different scales, and the hybrid encoder is a key innovation in the RT-DETR model, optimized for the traditional transformer’s inefficiency in handling multi-scale features. The hybrid encoder combines the strengths of convolutional neural networks (CNNs) and transformers, enhancing feature representation quality by decoupling intra-scale feature interaction and cross-scale feature fusion. The transformer decoder is another core component of the RT-DETR model, responsible for generating predictions such as object bounding boxes, categories, and embedding vectors based on the feature maps output by the hybrid encoder. Unlike traditional anchor-based detectors, RT-DETR adopts an end-to-end prediction approach, directly predicting object attributes through a set of learnable queries.

YOLO introduced a groundbreaking approach by framing object detection as a single regression problem, predicting bounding boxes and class probabilities directly from the entire image in one forward pass through the network. This made YOLO exceptionally fast compared to its predecessors, making it suitable for real-time applications. However, early versions of YOLO had limitations in detecting small and overlapping objects and struggled with localization accuracy [28].

These traditional algorithms laid the groundwork for the rapid advancements in object detection seen in later models. They highlighted the trade-offs between speed and accuracy and set the stage for more sophisticated approaches, such as the newer iterations of the YOLO series, which aim to overcome these limitations.

### 2.2. Development of the YOLO Series

The YOLO series has significantly influenced the field of object detection since its introduction [14]. By reimagining object detection as a single regression problem, YOLO enables real-time processing with relatively high accuracy, making it a groundbreaking approach in computer vision.

YOLOv1 laid the foundation by dividing an input image into a grid and predicting bounding boxes and class probabilities directly from each grid cell [14]. While this approach offered unprecedented speed, it struggled with detecting small and overlapping objects due to the limitations of its grid-based prediction method. In response to these challenges, YOLOv2 introduced anchor boxes, which improved the accuracy of bounding box predictions by allowing the model to predict multiple bounding boxes for each grid cell [29]. YOLOv2 also adopted the Darknet-19 backbone, significantly enhancing its feature extraction capabilities. The introduction of multi-scale training further allowed the model to generalize better across different object sizes and shapes, making YOLOv2 a more robust solution.

Furthermore, YOLOv3 incorporated a multi-scale feature pyramid network (FPN), which enabled better detection of objects at varying scales by merging features from different layers of the network [30]. YOLOv3 also improved class prediction accuracy, enhancing the model’s ability to recognize objects across a broader range of categories. YOLOv4 and YOLOv5 continued to refine the architecture with a focus on optimizing both speed and accuracy [31,32]. These versions integrated better backbone networks, more advanced feature fusion techniques, and improved loss functions. The enhancements made in these iterations further solidified YOLO’s position as a leading framework for real-time object detection, particularly in scenarios requiring a balance between computational efficiency and detection performance.

With the introduction of YOLOv8, the series saw significant architectural innovations, including the use of cross-stage partial network (CSPNet) for more efficient feature extraction and anchor-free detection heads that simplified the model’s design [33]. YOLOv8 also leveraged the SiLU activation function, which facilitated better gradient flow during training, leading to faster convergence and higher accuracy.

The most recent version, YOLOv10, represents a culmination of these advancements, introducing a non-maximum suppression (NMS)-free training strategy that minimizes inference delays [16]. YOLOv10 also employs a dual-label assignment mechanism that enhances both recall and precision, making it the most advanced and capable iteration in the YOLO series.

The continuous evolution of the YOLO series highlights the ongoing efforts to balance speed, accuracy, and efficiency, ensuring that each new version builds on the strengths of its predecessors while addressing their limitations.

### 2.3. Application of YOLO

The YOLO series has seen widespread adoption across various industries due to its real-time object detection capabilities. In autonomous driving, YOLO is used for detecting pedestrians, vehicles, and other road elements, ensuring timely decisions for safety [34]. In surveillance and security, it aids in monitoring environments, detecting intruders, and analyzing crowd behavior, making it ideal for real-time threat detection [35]. YOLO’s applications can also extend to healthcare, where it assists in detecting abnormalities in medical images [36]. In agriculture, YOLO is employed in precision farming to monitor crop health and detect pests, helping optimize yields [37]. In public service, YOLO is used to enhance automatic pavement distress recognition to assist highway maintenance decision making [38]. In the retail industry, YOLO powers automated checkout systems by accurately identifying products, enhancing customer experience, and streamlining inventory management [2]. The versatility and efficiency of YOLO across these diverse applications highlight its significant impact on real-time object detection across multiple sectors.

## 3. The Improved MidState-YOLO-ED Network

### 3.1. Integration of YOLOv8n and YOLOv10

One of the most distinct features of YOLOv10 compared to its predecessors is the elimination of non-maximum suppression (NMS), achieved by introducing a consistent dual-assignment strategy [16]. This strategy involves calling the loss function calculation method on YOLOv8n twice, summing the results, and returning them. The goal of this approach is to address the issue of redundant predictions in post-processing, aligning more closely with the end-to-end direction of the RT-DETR model. However, this modification resulted in less accuracy for many datasets in practical applications. To prevent a loss of precision, the prediction head of YOLOv10 was reverted back to that of YOLOv8n.

YOLOv10 introduces several modifications to enhance efficiency and reduce computational redundancy. These include a lightweight classification head, spatial-channel decoupled downsampling (SCDD), and a rank-based block design (i.e., C2fUIB). SCDD is a two-step process that first adjusts the channel dimensions using point-wise convolution, followed by spatial downsampling using depth-wise convolution. This reduces the number of parameters and helps to minimize information loss during the downsampling process. However, experiments have shown that some information loss still occurs, which can result in reduced latency but not necessarily improved performance.

This study modifies two core components of YOLOv10, reverting them back to their YOLOv8n counterparts and integrating both versions. As a result, the current model has been named MidState-YOLO. The model also incorporates efficient multi-scale attention (EMA) and the C2f-Dual convolution design, ultimately leading to the final model being named the MidState-YOLO-ED network.

### 3.2. Integration of EMA Attention

**Figure 1:**
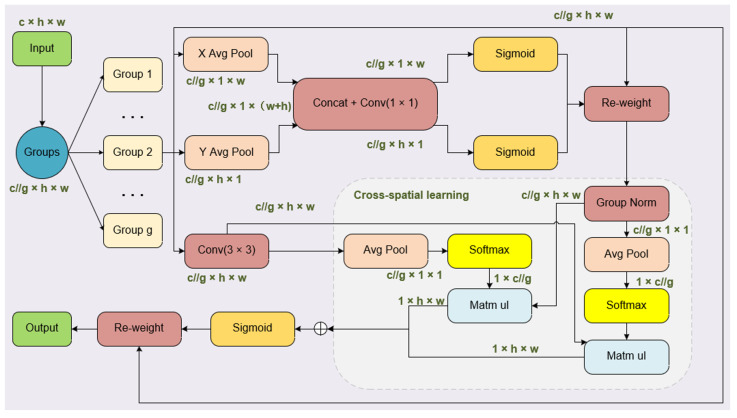
Integration of EMA.

To further enhance the expressive capability of the MidState-YOLO network and establish long and short dependency relationships, EMA has been integrated into the neck network. EMA is a parallel attention mechanism used in computer vision tasks to improve model performance and processing speed. Unlike traditional CNNs, EMA adopts a parallel structure to handle input data. This parallel convolution allows faster training of models when dealing with large-scale data and enhances accuracy by enabling parallel processing of features at different scales. In Figure 1, the divided groups are represented by “g”, while “X Avg Pool” denotes one-dimensional horizontal global pooling, and “Y Avg Pool” represents one-dimensional vertical global pooling, respectively. The formula for the average pooling operation is as follows, where *Xc*(*i*, *j*) represents the element at position (*i*, *j*):

(1) $$Z_{c}=\frac{1}{H\times W}\sum_{j}^{H}\sum_{i}^{W}X_{c}\left(i,j\right)$$

The input to EMA is first grouped and reshaped, redistributing part of the channel dimensions to the batch dimension. This is followed by further subdivision of the channel dimension into multiple sub-features to preserve key information in each channel and optimize the distribution of spatial semantic features [39].

This structure contains two main parallel branches: one branch performs one-dimensional global pooling operations to encode global information, while the other branch performs feature extraction through a 3 × 3 convolution. The output features from these two branches are modulated through a sigmoid function and normalization processes, then merged through a cross-dimensional interaction module to capture pixel-level pairwise relationships. Finally, the sigmoid-modulated output feature maps are used to enhance or weaken the original input features, thus achieving a more refined and effective feature representation. Therefore, EMA not only encodes inter-channel information to adjust the importance of various channels but preserves precise spatial structural details within these channels.

### 3.3. Lightweight Dual Convolution C2f-Dual Design

In YOLOv8 and YOLOv10, the C2f module integrates both low-level and high-level feature maps, facilitating the capture of gradient information flows. However, with the increasing number of layers in CNNs, semantic information in feature maps tends to be progressively extracted and aggregated, leading to redundancy in deep feature maps. Additionally, due to the weight-sharing mechanism of convolutional layers, convolutional kernel parameters are shared at different positions in the deep feature maps, further exacerbating redundancy. Bottleneck modules, composed of many complex convolutions, significantly increase parameter size and computational complexity.

**Figure 2:**
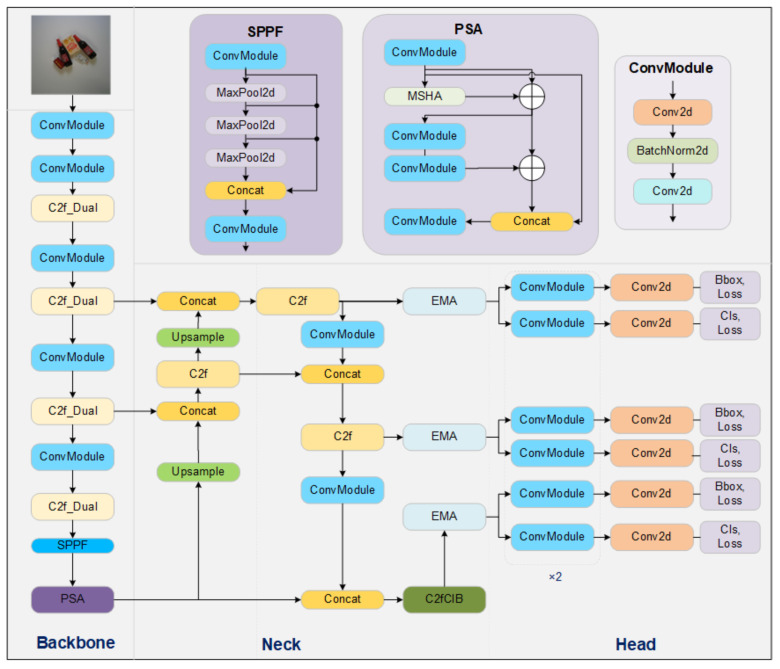
MidState-YOLO-ED.

To address this issue, the C2f-Dual convolution design, improved using dual convolutional kernels (DualConv), significantly reduces computational costs and the number of parameters while also enhancing precision. This improvement involves replacing the C2f modules before the spatial pyramid pooling fast (SPPF) with C2f-Dual modules, as shown in Figure 2. This adaptation not only streamlines the network but also optimizes performance by ensuring that critical spatial and semantic features are efficiently processed and integrated.

DualConv is designed to build lightweight deep neural networks by combining 3 × 3 and 1 × 1 convolution kernels to process the same input feature map channels, optimizing information processing and feature extraction. In DualConv, the 3×3 convolution kernels are used to extract spatial features from the feature maps, capturing more spatial information, while the 1×1 convolution kernels integrate these features and reduce the model’s parameters. Each group of convolution kernels processes a portion of the input channels independently before the outputs are merged, facilitating efficient flow and integration of information across different feature map channels.

Additionally, DualConv employs group convolution technology to efficiently arrange convolution filters. In group convolution, both input and output feature maps are divided into multiple groups, with each group’s convolution filters processing only a part of the corresponding input feature map. This arrangement allows different kernels within a group to process the same set of input channels in parallel, optimizing information flow and feature extraction efficiency while maintaining the network’s representational capabilities.

Thus, replacing the bottleneck structures in C2f with DualBottleneck enriches gradient flow representation, enhances feature extraction capabilities, and reduces the diversity of false positives and false negatives in network learning. This makes it more suitable for retail commodity object detection scenarios.

## 4. Experimental Results and Analysis

### 4.1. Experimental Setup and Parameters

The hardware environment and software configurations used for the experiments are listed in Table 1. During the model training process, the learning rate was set to 0.01, with optimization carried out using stochastic gradient descent (SGD). The momentum parameter was set at 0.937, and the weight decay factor was 0.0005. The batch size used was 32, and the image size was 640×640 pixels. The comparative experiments were conducted over 30 epochs, while the ablation studies were carried out over 25 epochs.

### 4.2. Dataset

This study employs a portion of the retail product (RPC) dataset for training and validation. The RPC dataset, developed by Megvii Technology’s Nanjing Research Institute, is currently the largest product recognition dataset available [40]. It includes up to 200 different product categories and a total of 83,000 images, realistically simulating retail environments and surpassing existing datasets in fidelity. Moreover, it effectively captures the fine-grained characteristics inherent in the automatic check-out (ACO) problem.

**Figure 3:**
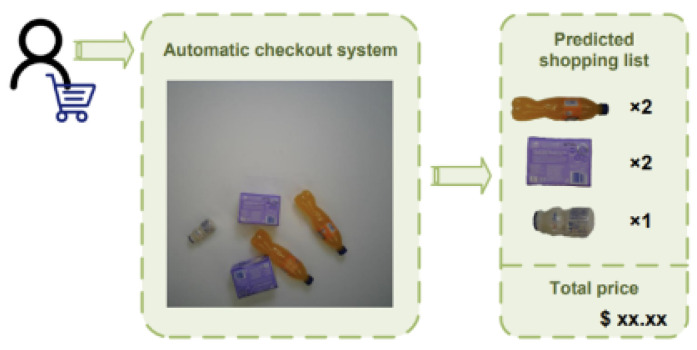
Dual convolution C2f-Dual design.

The conceptual approach of this study may differ from that of the researchers who collected the RPC dataset. When customers enter a store and place the items they wish to purchase on the checkout counter, an ideal ACO system would automatically identify each product and accurately generate a shopping list in one go, as shown in Figure 3. Thus, ACO is fundamentally a system designed to identify and count the occurrence of each item in any combination of products.

This research posits that there are multiple important metrics for assessing performance on ACO tasks. To ensure accuracy and performance, the images used to train the ACO recognition system should mirror the actual retail checkout environments, which can indeed be simplified and stabilized. Additionally, initial models do not need to exhaust all product combinations to perform ACO tasks; instead, creating random groups of product combinations suffices.

Therefore, for this study, only the checkout configurations from the RPC dataset images are used. The single-product images are not included in the training dataset because they do not perfectly mimic real-life retail scenarios. As a result, only 30,000 out of the 83,000 images from the RPC dataset are utilized for the research. We randomly divided 30,000 images of checkout configurations into training, validation, and test sets in an 8:1:1 ratio. This approach aims to provide a realistic yet controlled set of data that reflects real-world ACO system operations while maintaining manageable complexity and variety in training scenarios. The dataset includes checkout images with varying levels of clutter, capturing the real-world challenge of recognizing products when multiple items are grouped together in a single scene. However, further research will focus on addressing different lighting conditions, either by utilizing additional datasets or through image augmentation techniques. By doing so, it could help reduce potential biases inherent in the dataset, leading to a more robust and generalized model.

### 4.3. Evaluation Metrics

This paper employs precision (*P*), recall (*R*), mean average precision (*mAP*), number of parameters (Params), floating point operations (FLOPs), and frames per second (FPS) as evaluation metrics, with a set IoU threshold of 0.5. Note that mAP@0.5 denotes the mean average precision when the IoU is set at 0.5, and mAP@0.5:0.95 indicates the mean average precision when the IoU ranges from 0.5 to 0.95, with a step size of 0.05. The floating point operations indicate the complexity of the algorithm. The specific meanings of other performance metrics are as follows:

(2) $$P=(\frac{TP}{TP+FP})\times 100$$

(3) $$R=(\frac{TP}{TP+FN})\times 100$$

where precision is the probability that a positive sample predicted by the model is indeed a positive sample, and recall is the probability that a positive sample in reality is predicted as positive by the model. There are two important metrics used to evaluate model performance, AP and mAP, such that:

(4) $$AP=\int_{0}^{1}p\left(r\right)dr$$

(5) $$mAP=\frac{1}{n_{j}\sum_{j=1}^{n_{j}}AP_{j}}$$

In Formulas (2) and (3), *TP* (true positives) denotes positive examples correctly identified as positive by the model; *FP* (false positives) denotes negative examples incorrectly identified as positive; and *FN* (false negatives) denotes positive examples incorrectly identified as negative.

In Formulas (4) and (6), *mAP* represents the mean of the average precision across all object detection categories. AP is the average of precision values at different recall levels. The curve plotted with precision as the y-axis and recall as the x-axis is known as the PR curve. *mAP* can be calculated as the average area under the PR curves for all categories, *n* represents the number of instances in a given category, and *APj* represents the detection precision for category *j*.

FPS is an important metric for measuring the speed of a model’s image processing capability. It indicates the number of images the model can detect in one second, determining the model’s response speed and real-time performance in practical applications. The higher the FPS value, the more images the model can process within a unit of time, thus indicating a faster detection speed. Specifically:

(6) $$FPS=\frac{FrameNum}{ElapsedTime}$$

where *FrameNum* is the total number of images detected, and *ElapsedTime* is the total time the model took to perform the detection.

### 4.4. Ablation Study

To investigate the extent of improvements from the three modification schemes, ablation studies were conducted using YOLOv8-n and YOLOv10-n as baseline networks. These experiments were carried out on the experimental dataset without changing the software and hardware environment, with the only parameter change being the reduction of epochs to 25. As indicated in Table 2, the MidState-YOLO network, which integrates modules from YOLOv8 and YOLOv10, achieved a 23.2% increase in mAP compared to the original baseline network of YOLOv10-n. This suggests that combining two models can harness the advantages of both while avoiding some of their respective shortcomings, thereby enhancing model performance.

Using the lightweight dual convolution C2f_Dual module in the MidState-YOLO network significantly reduced the number of parameters, and all performance metrics showed improvements. The reason for this is the reduction in redundant information in the deep feature maps and a decrease in false positives and false negatives during network learning. The inclusion of the EMA module increased the mAP@0.5 to 84.4%, resulting in a 4.2% gain. This demonstrates that the EMA module effectively captures global information to learn richer semantic features, focusing the network model more on the overall context of retail product targets and enhancing model performance.

The final improved model, MidState-YOLO-ED, showed improvements in all evaluation metrics relative to YOLOv8-n, with precision and recall increasing by 2.3 and 1.6 percentage points, respectively, and *mAP* reaching 89%. Additionally, the number of parameters and floating point operations were significantly lower.

### 4.5. Experimental Results and Discussion

The experimental results primarily organize the performance parameters of the trained algorithms, explaining the strengths and weaknesses of each algorithm based on these results, and analyzing the experimental data and actual detection effects. All experiments were conducted under the same configuration settings, and after training, the weight files generated by each algorithm were tested. The algorithms used in the comparative experiments include SSD, Faster-RCNN, YOLOv8-n, YOLOv10-n, and RT-DETR-L. The experimental results are shown in Table 3.

The experimental results indicate that, compared to the SSD and Faster-RCNN algorithms, the YOLO series algorithms and the improvements introduced in this study exhibit superior detection performance. Additionally, since Faster-RCNN is a two-stage algorithm, its applicability is limited by its complexity and the extended duration required for detection, which makes its overall performance inferior to that of the YOLO series.

The comparative results of different algorithms demonstrate that the MidState-YOLO-ED algorithm excels in terms of the number of parameters and floating point operations, with only 3,288,096 parameters and 9.6 GFLOPs. This fully proves the excellent performance of the improved algorithm in terms of lightweight design. The algorithm can process image data quickly and accurately, making it suitable for scenarios requiring fast response. It is also more appropriate for operation in resource-constrained environments such as mobile devices and embedded systems, and it meets real-time requirements [41]. As a current mainstream real-time object detection model, the RT-DETR algorithm performs excellently in terms of accuracy. However, it has similar issues to the Faster-RCNN algorithm, such as a large number of parameters and suboptimal time performance in retail product detection.

Speed tests were conducted on different detection algorithms to evaluate their detection efficiency. The speed tests were conducted in the same GPU environment, and the results showed that the improved MidState-YOLO-ED algorithm surpassed most algorithms in detection speed, achieving an impressive improvement of 71.88 fps compared to the Faster-RCNN algorithm.

Furthermore, key indicators such as recall and mAP for the MidState-YOLO-ED algorithm are higher than those of baseline algorithms, offering a new option for efficient and rapid object detection.

**Figure 4:**
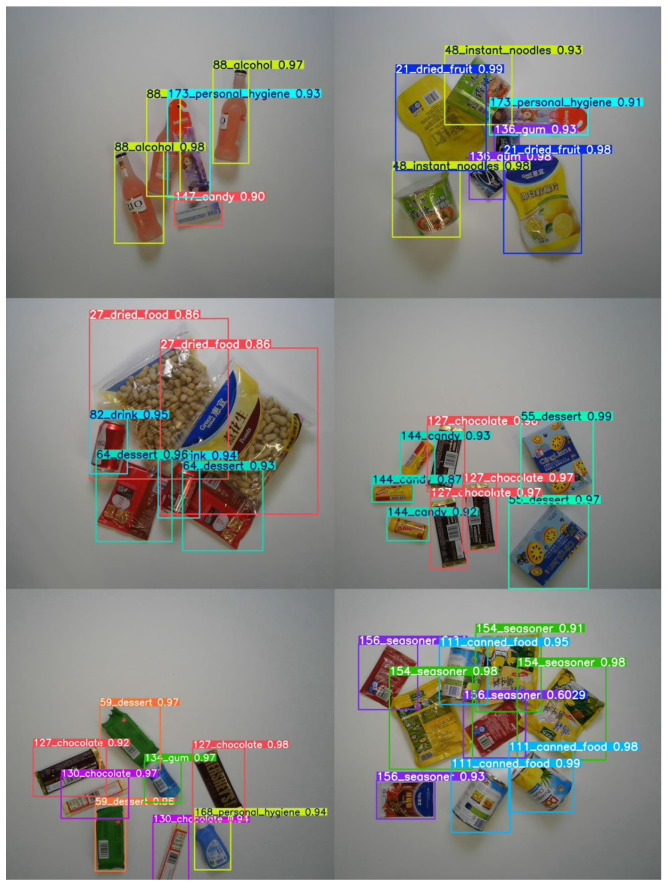
Prediction results.

After training the final improved model, MidState-YOLO-ED, for 30 epochs, the prediction results are displayed in Figure 4. The results demonstrate that the algorithm proposed in this paper achieves excellent detection performance while maintaining a lightweight framework.

### 4.6. Comparative Visualization Analysis

**Figure 5:**
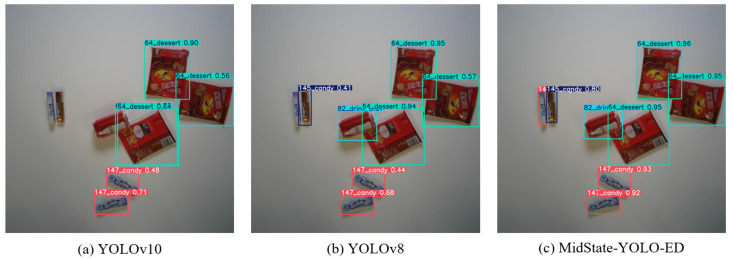
Comparative visualization analysis.

To visually illustrate the effectiveness and superiority of MidState-YOLO-ED, a set of images was selected for testing using YOLOv8-n, YOLOv10-n, and the proposed model. The detection results are shown in Figure 5, where Figure 5a–c in the figure represent the test results of the YOLOv10, YOLOv8, and MidState-YOLO-ED models, respectively. It is clear from the figure that both the YOLOv10 and YOLOv8 models perform poorly in the retail product detection environment, resulting in missed detections. In Figure 5a, although the distribution of products is not dense, the YOLOv10 model still detects a single product as multiple instances, which is a critical issue in the retail product detection field. Figure 5c clearly shows that MidState-YOLO-ED can effectively avoid a series of problems present in the original models.

### 4.7. Loss Function Analysis

**Figure 6:**
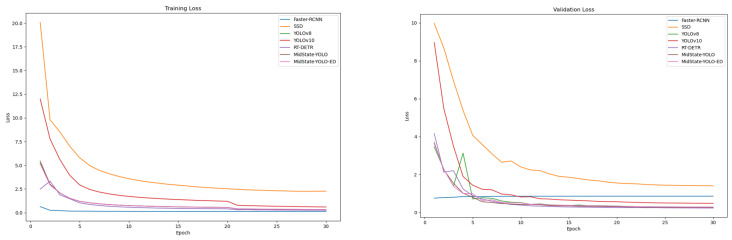
Loss function analysis.

Based on the logs saved during the training process, loss comparison curves for seven models were plotted. Figure 6 (left) represents the training loss, while Figure 6 (right) represents the validation loss. The horizontal and vertical axes represent the number of epochs and the loss value, respectively. For most models, both training loss and validation loss steadily decrease as the training progresses. The loss of the Faster-RCNN model begins to rise after reaching a certain low point, indicating that the Faster-RCNN model is too complex for the retail environment and has a risk of overfitting. Observing the proposed MidState-YOLO-ED model, it demonstrates good stability, with its validation loss remaining relatively stable throughout the training cycle without significant fluctuations. This indicates that the MidState-YOLO-ED model is able to effectively learn data features during training while maintaining good generalization capability, which is crucial for object detection tasks in practical applications.

## 5. Discussion

The improvements proposed in our system, particularly the integration of efficient multi-scale attention and the C2f-Dual convolution module, directly contribute to reducing these errors by enhancing both spatial and contextual feature extraction. This results in better differentiation between visually similar products, such as different flavors of chocolate or sizes of packaged goods, which are typically challenging for conventional object detection models.

However, our study has limitations. Currently, our system does not explicitly process a full 360-degree rotation of objects. However, the model’s capability to handle diverse viewpoints is enhanced by the use of multi-scale attention mechanisms and augmented data during training. These components help improve the model’s generalization to various angles, including side views and tilted projections. To address the need for 360-degree object recognition more thoroughly, future work could incorporate multi-view learning or synthetic data generation to simulate a wider range of object orientations. This would allow the model to learn how to recognize objects from all possible angles, which is particularly important for products that may be placed on the checkout surface in unpredictable orientations.

## 6. Conclusions

In this paper, we presented an enhanced self-checkout system using an improved YOLOv10 network. The system significantly advances retail automation by optimizing checkout efficiency and minimizing labor costs. Our adaptations to the YOLOv10 model, integrating features from YOLOv8 and new post-processing algorithms, markedly improves product recognition accuracy, with our experiments demonstrating superior performance over existing systems.

Broader applications in inventory control and customer service will be benefited by this study. Our study shows that AI-driven technologies will play a pivotal role in enhancing consumer experiences and operational efficiency.

## Figures and Tables

**Table 1:** Hardware and software configuration for experimental environment.

Configuration	Parameter
GPU	RTX4080-16G
CPU	AMD Ryzen7
Operating System	Windows11
Deep Learning Frameworks	Pytorch2.1.1
	+cuda12.1
Build System	PyCharm

**Table 2:** Ablation study experiments with improved strategies.

Model	Precision	Recall	mAP@0.5	mAP@0.5:0.95	Params	GFLOPs
YOLOv8-n	0.824	0.809	0.877	0.691	3,371,024	9.8
YOLO v10-n	0.551	0.595	0.61	0.481	**2,885,888**	**9.2**
MidState-YOLO	0.794	0.775	0.842	0.654	3,405,456	9.8
MidState-YOLO+DualConv	0.84	0.816	0.883	0.686	3,251,856	9.4
MidState-YOLO+EMA	0.843	0.813	0.884	**0.694**	3,408,928	9.9
MidState-YOLO-ED	**0.847**	**0.825**	**0.89**	0.691	3,288,096	9.6

**Table 3:** Comparison of experimental data.

Model	Recall	mAP@0.5	mAP@0.5:0.95	Params	GFLOPs	FPS
Faster R-CNN	0.899	**0.995**	0.855	41,808,406	134.9	38.01
SSD	0.758	0.943	0.693	30,160,468	13.4	58.78
YOLOv8-n	0.981	0.992	0.869	3,371,024	9.8	106.38
YOLO v10-n	0.970	0.991	0.871	**2,885,888**	**9.2**	**112.36**
RT-DETR-L	**0.991**	0.994	**0.879**	32,394,740	104.3	89.29
MidState-YOLO	0.987	0.993	0.875	3,405,456	9.8	105.26
MidState-YOLO-ED	0.985	0.994	0.875	3,288,096	9.6	109.89

## Data Availability

The data included in this manuscript cannot be shared publicly, due to the need to protect the privacy of the included subjects. Data may be shared upon reasonable request to the corresponding author.
